# CNS tumor with *EP300::BCOR* fusion: discussing its prevalence in adult population

**DOI:** 10.1186/s40478-023-01523-y

**Published:** 2023-02-13

**Authors:** Arnault Tauziède-Espariat, Emmanuelle Uro-Coste, Philipp Sievers, Yvan Nicaise, Cassandra Mariet, Aurore Siegfried, Gaëlle Pierron, Delphine Guillemot, Joseph Benzakoun, Johan Pallud, Margaux Roques, Fabrice Bonneville, Delphine Larrieu-Ciron, Patrick Chaynes, Raphaël Saffroy, Jocelyne Hamelin, Lauren Hasty, Alice Métais, Fabrice Chrétien, Marcel Kool, Johannes Gojo, Pascale Varlet, RENOCLIP-LOC

**Affiliations:** 1 Department of Neuropathology, GHU Paris - Psychiatry and Neuroscience, Sainte-Anne Hospital, 1, Rue Cabanis, 75014 Paris, France; 2 Institut de Psychiatrie et Neurosciences de Paris (IPNP), UMR S1266, INSERM, IMA-BRAIN, Paris, France; 3 Université de Paris, Paris, France; 4 Department of Pathology, Toulouse University Hospital, Toulouse, France; 5 Cancer Research Center of Toulouse (CRCT), INSERM U1037, Toulouse, France; 6 Université Paul Sabatier, Toulouse III, Toulouse, France; 7 Department of Neuropathology, Institute of Pathology, University Hospital Heidelberg, Heidelberg, Germany; 8 Clinical Cooperation Unit Neuropathology, German Cancer Research Center DKFZ), German Consortium for Translational Cancer Research (DKTK), Heidelberg, Germany; 9 Paris-Sciences-Lettres, Curie Institute Research Center, INSERMU830 Paris, France; 10 Laboratory of Somatic Genetics, Curie Institute Hospital, Paris, France; 11 Department of Radiology, GHU Paris-Psychiatrie et Neurosciences, Sainte-Anne Hospital, Paris, France; 12 Department of Neurosurgery, GHU Paris-Psychiatrie et Neurosciences, Sainte-Anne Hospital, Paris, France; 13 Department of Radiology, Purpan University Hospital, Toulouse, France; 14 Department of Neurology, Toulouse University Hospital, Toulouse, France; 15 Department of Medical Oncology, IUCT-Oncopole, Toulouse, France; 16 Department of Neurosurgery, Toulouse University Hospital, Toulouse, France; 17 Department of Biochemistry and Oncogenetic, Paul Brousse Hospital, Villejuif, France; 18 Hopp Children’s Cancer Center (KiTZ), Heidelberg, Germany; 19 Division of Pediatric Oncology, German Cancer Research Center (DKFZ) and German Cancer Consortium (DKTK), Heidelberg, Germany; 20 Princess Máxima Center for Pediatric Oncology, Utrecht, The Netherlands; 21 Department of Pediatric and Adolescent Medicine, Comprehensive Center for Pediatrics and Comprehensive Cancer Center, Medical University of Vienna, Vienna, Austria

**Keywords:** EP300, BCOR, Adult

## Abstract

The Central Nervous System (CNS) tumor with *BCOR* internal tandem duplication (ITD) has recently been added as a novel embryonal histomolecular tumor type to the 2021 World Health Organization (WHO) Classification of CNS Tumors. In addition, other CNS tumors harboring a *BCOR/BCORL1* fusion*,* which are defined by a distinct DNA-methylation profile, have been recently identified in the literature but clinical, radiological and histopathological data remain scarce. Herein, we present two adult cases of CNS tumors with *EP300::BCOR* fusion. These two cases presented radiological, histopathological, and immunohistochemical homologies with CNS tumors having *BCOR* ITD in children. To compare these tumors with different *BCOR* alterations, we performed a literature review with a meta-analysis. CNS tumors with *EP300::BCOR* fusion seem to be distinct from their *BCOR* ITD counterparts in terms of age, location, progression-free survival, tumor growth pattern, and immunopositivity for the BCOR protein. CNS tumors from the *EP300::BCOR* fusion methylation class in adults may be added to the future WHO classification.

**Supplementary Information:**

The online version contains supplementary material available at 10.1186/s40478-023-01523-y.

## Introduction

The current version of the World Health Organization’s (WHO) Classification of Tumors of the Central Nervous System (CNS) identified a novel embryonal histomolecular type, the “CNS tumor with *BCOR* internal tandem duplication”[1]. These tumors, characterized by a recurrent internal tandem duplication (ITD) of the *BCOR* (*BCL6 Corepressor*) gene [2–17], are almost exclusively found in young children and remain a diagnostic and therapeutic challenge [18]. Recently, cases of CNS tumors harboring *EP300::BCOR(L1)* fusions (21 reported cases) were described, sharing histopathological features with CNS tumors having *BCOR* ITD, but not the same methylation class [5, 17, 19]. However, very little data concerning clinical presentation, radiology and detailed histopathology are available in the literature. Herein, we report two other CNS tumors with *EP300::BCOR* fusion affecting adults. We summarize their clinical, histopathological, immunophenotypical, genetic and epigenetic features as compared to reports on tumors with *BCOR* ITD, based on analysis of previously reported cases.

## Case presentation

**Fig. 1:**
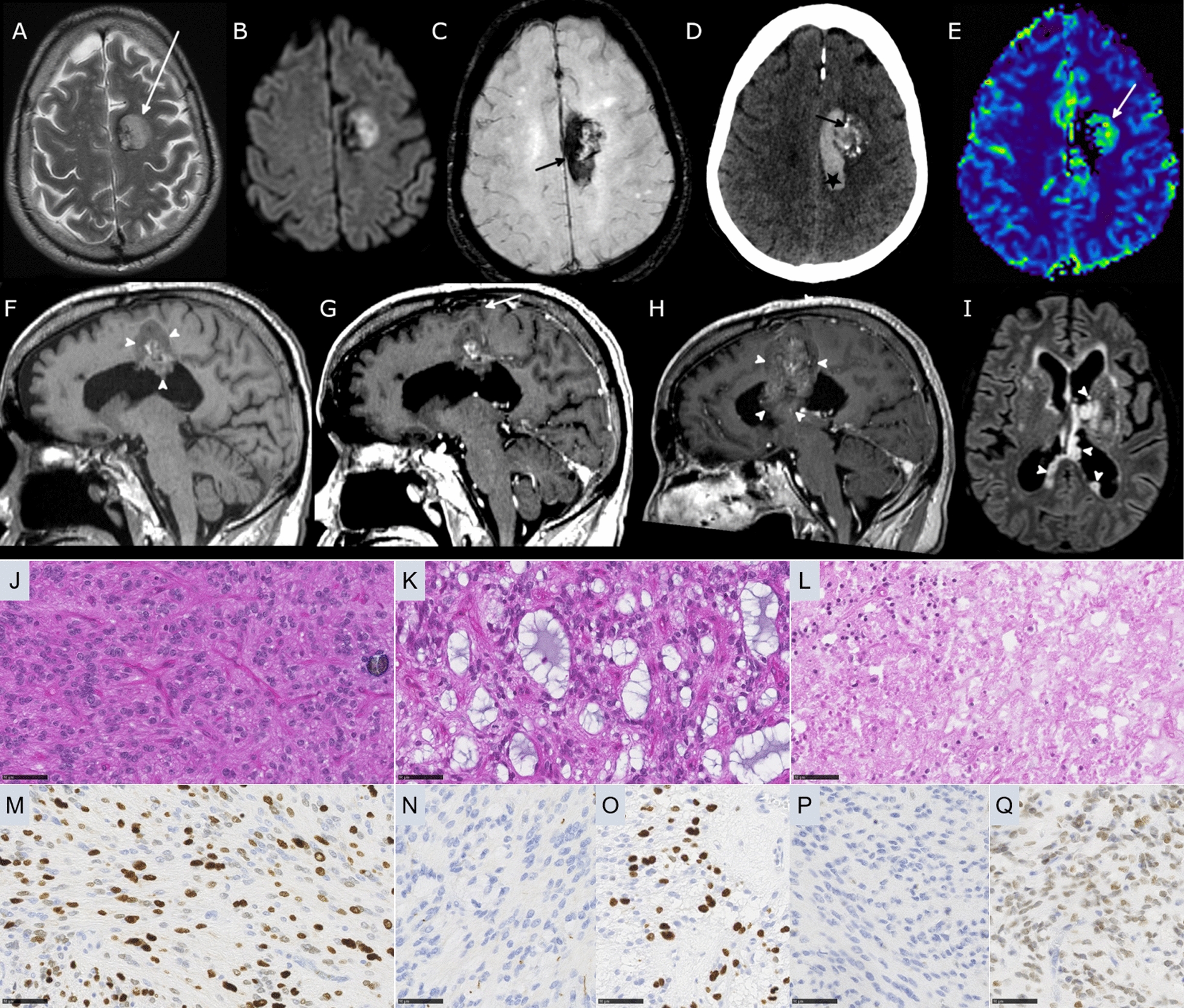
Radiological features of Case #1 tumor. **a** Axial T2-weighted MRI sequence showing a well-delineated left frontal hyperintense lesion (arrow). **b** Axial diffusion-weighted sequence demonstrating hyperintensity corresponding to hypercellularity of the solid component **c** Axial susceptibility-weighted sequence showing hypointensity consistent with hemorrhage within the tumor (arrow). **d** Axial CT showing calcifications (arrow) and acute hemorrhage (star) **e** Axial perfusion sequence with Cerebral Blood Volume Map showing moderate hyperperfusion of the solid component (arrow). Sagittal T1-weighted **f** before and **g** after contrast injection demonstrate juxta-ventricular location of the mass lesion (arrowheads) and a subtle area of enhancement (arrow) **h** MRI follow-up at two years with post-contrast sagittal T1-weighted sequence showing tumor progression and increase of enhanced components. **i** Axial FLAIR sequence performed one year after surgery showed an intraventricular ependymal dissemination (arrowheads). **j** Ependymal differentiation with calcifications (HPS, magnification 400 ×). **k** Microcysts containing myxoid substance (HPS, magnification 400 ×). **l** Necrosis (HPS, magnification 400 ×). **m** Olig2 immunoreactivity (magnification 400 ×). **n** No immunoexpression for GFAP (magnification 400 ×). **o** MIB1 labeling index (magnification 400 ×). **p** No immunopositivity for BCOR (magnification 400 ×). **q** Expression of SATB2 (magnification 400 ×) Black scale bars represent µm. *HPS* Hematoxylin Phloxin Saffron.

**Fig. 2:**
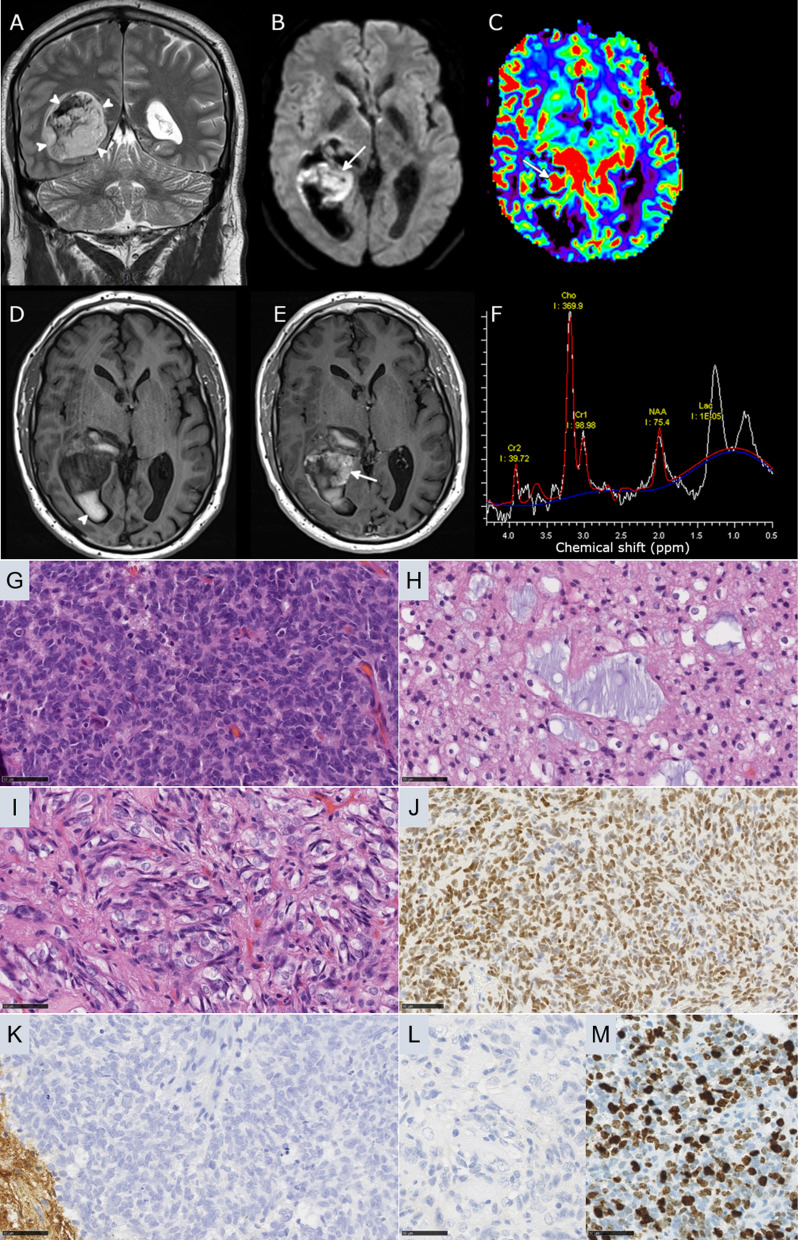
Radiological features of Case #2 tumor. **a** Coronal T2-weighted MRI sequence showing a well-delineated tumor developed inside the right ventricle. **b** Axial diffusion-weighted sequence showing hyperintensity consistent with hypercellularity of the solid component (arrow) **c** MR Perfusion with Cerebral Blood Volume Map showing hyperperfusion of the tumor (arrow). **d** Axial T1-weighted injection showing a spontaneous hyperintense intra-ventricular haemorrhage (arrowhead); **e** strong enhancement of the solid component is present after contrast injection (arrow) **f** MR Spectroscopy demonstrated elevated Choline (Cho), decreased NAA and elevation of lipids-lactates complex (Lac). **g** Ependymal differentiation (HPS, magnification 400 ×). **h** Microcysts containing myxoid substance (HPS, magnification 400 ×). **i** Spindle cell component (HPS, magnification 400 ×). (**J**) Olig2 immunoexpression by tumor cells (magnification 400 ×). **k** No immunopositivity for GFAP (magnification 400 ×). **l** MIB1 labeling index (magnification 400 ×). **m** No immunopositivity for BCOR (magnification 400 ×). Black scale bars represent µm. *HPS* Hematoxylin Phloxin Saffron

The two observed cases concerned a 64-year-old man (Case #1, Fig. 1) with a history of radiation therapy for a nasal tumor discovered 40 years ago, and a 45-year-old man (Case #2, Fig. 2), with an initial clinical presentation of intraventricular hemorrhage. A central neuroradiological review showed both tumors to be well-circumscribed and hemorrhagic. In Case #1, the tumor was located in the periventricular area of the left frontal lobe, and in Case #2, the tumor was within the right lateral ventricle. Both lesions had a peripheral solid hypercellular portion appearing hyperintense on T2-weighted images with a restricted apparent diffusion coefficient (ADC). The solid component contained a weakly enhancing area in Case #1 at baseline, and a strongly enhancing portion in Case #2. Both presented a heterogeneous center due to recent hemorrhage. No significant peripheral parenchymal edema was present. Nodular peripheral calcifications were present in Case #1; calcifications were not evaluable in Case #2. Magnetic resonance spectroscopy analysis demonstrated elevated choline and the presence of lipids in both cases. Magnetic resonance perfusion revealed hyperperfusion of the solid components. In Case #1, follow-up imaging showed a regression of the intratumoral hemorrhage, and a slow tumor progression with an increase of enhancing components and the apparition of a central necrotic core.

**Fig. 3:**
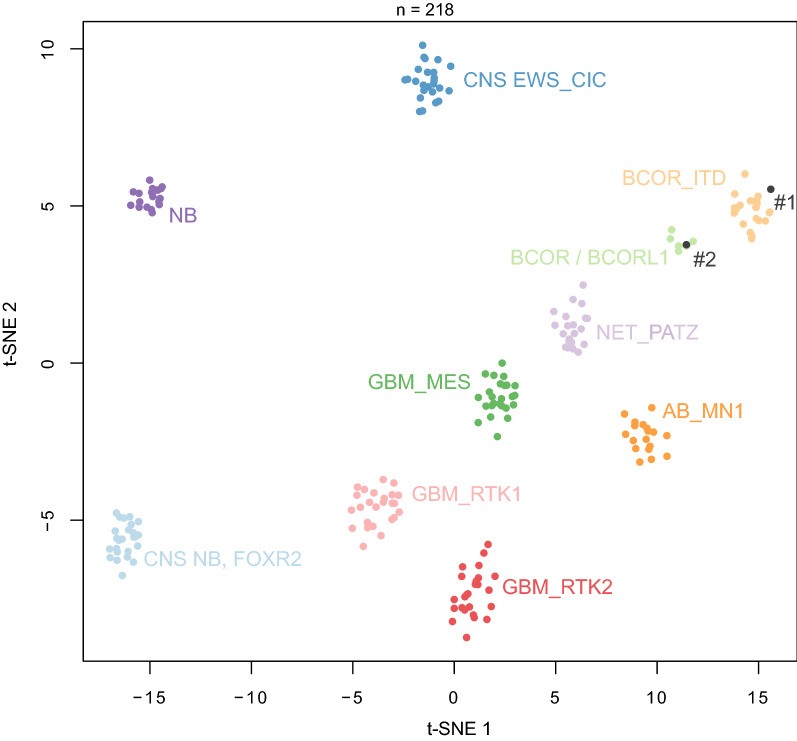
Methylation-based t-SNE distribution. Reference DNA methylation classes (v12.5 of the DKFZ classifier): AB_MN1: Astroblastoma, MN1-altered, MN1:BEND2-fused; BCOR / BCORL1: CNS tumor with BCOR/BCORL1 fusion; BCOR_ITD: CNS tumor with BCOR internal tandem duplication; CNS EWS_CIC: CIC-rearranged sarcoma; CNS NB, FOXR2: central nervous system neuroblastoma, FOXR2-activated; GBM_MES: Glioblastoma, IDH-wildtype, mesenchymal subtype; GBM_RTK1: Glioblastoma, IDH-wildtype, RTK1 subtype; GBM_RTK2: Glioblastoma, IDH-wildtype, RTK2 subtype; NB: neuroblastoma; NET_PATZ: Neuroepithelial tumor with PATZ1 fusion

Histopathological review revealed that both tumors presented similar features (Figs. 1 and 2). These tumors were mainly well-circumscribed from the brain parenchyma. There was intra-tumoral heterogeneity in terms of cytology, with oligo-like, embryonal, or ependymal features. Microcysts containing a myxoid substance were constantly observed. Both cases presented calcifications. Malignancy was obvious with palisading necrosis, a high mitotic count and proliferation index, but no microvascular proliferation was observed. Using immunohistochemistry, the tumor cells diffusely expressed Olig2 but were immunonegative for GFAP. They were well-circumscribed by neurofilament staining. Expression of at least one neuronal marker (neurofilament or NeuN) was present in both cases. There was no immunoreactivity for IDH1R132H, p53, or BCOR (Santa Cruz; Clone C-10) and there was a preserved expression of H3K27me3, and ATRX in both cases. An initial diagnosis of high-grade glioneuronal tumor was made for both cases. Next Generation Sequencing analysis failed to reveal any mutation in the *IDH1/2, BRAF, H3F3A, HIST1H3B* or *hTERT* genes. FISH analyses revealed a disomy for chromosomes 7 and 10. An RNA sequencing analysis was performed and showed the presence of a fusion between the *EP300* and *BCOR* genes, with intra exonic breakpoints (in exon 31 for *EP300*, and exon 4 for *BCOR*), for both cases. The DNA-methylation profiles, obtained using the Heidelberg DNA methylation classifier (v12.5), and t-Distributed Stochastic Neighbor Embedding (t-SNE) analysis classified them as a neuroepithelial tumor with *BCOR* ITD (with a calibrated max-score of 0.82 for Case #1), and a CNS tumor with a *EP300::BCOR(L1)* fusion (calibrated score of 0.99 for Case #2) (Fig. 3 and Additional file 1: Figure S1 for copy number variations plot). For Case #1 a gross total resection of the tumor was performed followed by a close follow-up: at one year postoperatively, the patient was alive with a progression of the tumoral residue and an ependymal dissemination of the tumor (Fig. 1). For Case #2, a partial resection of the tumor was performed followed by a chemotherapy (carboplatin and VP16) without radiation therapy. At the last follow-up, the patient was alive (one year after the initial surgery) with a progression of the tumoral residue and an ependymal dissemination of the tumor (Fig. 1).

### Discussion and conclusions

Herein, we describe two novel cases of *EP300::BCOR*-fused tumors in adult patients. To compare these cases to published data we performed an extensive review of existing literature. Based on our literature review, the median age of patients reported to suffer from CNS tumors *EP300::BCOR-*fused was 30 years-old (varying from 5 to 72 years old, with 70% of cases being adults) [5, 17, 19]. In summary, this is different from reported *BCOR* ITD cases where 94% are pediatric with a median age of 4 years-old at diagnosis, with extremes varying from 0 to 36 [2–15]. Particularly, three cases with CNS tumors *EP300::BCOR-*fused were older than 60 years of age [5, 17, 19]. In reported cases with *EP300::BCOR* fusion, patients were predominantly male whereas no sex predominance was observed in cases with ITD (sex ratio male/female: 1.55 *vs*. 1.0) [2–15, 17, 19]. As previously reported [17], CNS tumors with *EP300::BCOR* fusion are more frequently reported in the supratentorial region (83%, 15/18) than cases with *BCOR* ITD (56%, 42/75) [2–15, 17, 19]. Radiologically, the tumors with *EP300::BCOR* fusion described here shared similar features with *BCOR* ITD [20] (solid and hypercellular masses with a heterogeneous enhancement after a contrast injection, and the presence of calcifications in 1 of our 2 cases). Based on this preliminary observations, the tumors with *EP300::BCOR* fusion seem to be more frequently intra/juxta-ventricular than *BCOR* ITD tumors [20]. Histopathologically, no clear differences between CNS tumors with *BCOR* fusion or ITD have been described pointing towards the necessity of further in depth analyses in larger patient collectives. The tumors with BCOR fusions consist of highly cellular tumors with an alternance of microcystic, pseudo-ependymal, and oligodendroglioma-like pattern. However, CNS tumors with *BCOR* ITD [2–4, 6, 8, 10, 12, 15] seem to be more circumscribed than their counterparts having a *BCOR* fusion (n = 41/47 and n = 4/8 respectively) [17, 19]. Tumor cells are monotonous, round to oval with frequent mitoses and a high MIB1 labeling index. Palisading necrosis is frequent, but no microvascular proliferation is observed, which represents a distinctive element from high-grade gliomas. The immunophenotype may be particular with an expression of Olig2 without immunoreactivity for GFAP, independently of the *BCOR* alteration. This also represents a discriminating criterion from ependymal tumors. Neuronal markers (such as NeuN or NF70) are constantly observed. In contrast to 100% of reported CNS tumors with *BCOR* ITD, tumors with *BCOR* fusion do not all exhibit an overexpression of the BCOR protein by immunohistochemistry (present in 43% of reported cases) [17, 19, 21]. This discrepancy may be related to the use of different clones of antibodies. In detail, the antibody used in this study (BCOR santa crzs C-10) detects the c-terminal part of the BCOR protein which is lost in the EP300::BCOR fusion. Like tumors with ITD, potential differential histopathological diagnoses may include meningiomas, ependymomas, high-grade gliomas (particularly in adults), astrocytoma, *IDH-*mutant and oligodendroglioma, *IDH-*mutant with co-deleted 1p19q. The pronounced delineation of the tumor, the morphology, the immunohistochemical findings and the molecular features allow us to easily effectuate those diagnoses. The inconstant immunopositivity for BCOR constitutes a supplemental difficulty when suggesting the diagnosis for the CNS tumor with *BCOR* fusion in adults. A previous study evidenced the SATB2 biomarker to be sensible but not specific for diagnosing CNS tumors with *BCOR* alterations [21]. Consequently, RNA-sequencing analysis (detecting the presence of the fusion) or DNA-methylation profiling are needed to confirm the diagnosis. However, these techniques may also present their limitations. Indeed, as one case from the current study and some previously reported cases, there is no perfect correlation between the genetic alteration of *BCOR* and the methylation class. A subset of tumors with *EP300::BCOR* fusion are classified with a high calibrated score as CNS neuroepithelial tumor with *BCOR* ITD [17, 19]. Moreover, it seems like alternative fusions (*CREBBP::BCOR* and *MEAF6::CXXC5*) may be encountered as a subset of CNS tumors within the EP300::BCOR(L1) methylation class [17]. Finally, these tumors presenting histopathological similarities to their pediatric counterparts with *BCOR* ITD may be distinguished from a group of pediatric diffuse gliomas harboring fusions of *BCOR(L1)* genes (particularly in combination with *CREBBP*) but which did not cluster within the same methylation class [23–25].

In terms of prognosis, CNS tumors with *BCOR* ITD present a significantly higher rate of recurrences (65% of reported cases; with a median progression-free survival of 8 months, varying from 2 to 108 months after the initial diagnosis)[2–12, 14, 15] compared to CNS tumors with *BCOR(L1)* fusion (53% of reported cases; progression-free survival of 24.6 months, varying from 1 to 86 months), [5, 17, 18]. However, difference between the two tumor groups in terms of overall survival is not as pronounced (median overall survival of 38 months, varying from 2 to 170 months after the initial diagnosis for *BCOR* ITD) [2–15]. For tumors with *BCOR(L1)* fusion, the data are scarce (n = 5) but the patients studied are currently still alive [5, 18].

In conclusion, we present two novel cases of the rare CNS tumor having an *EP300::BCOR* fusion in adults, representing a distinct subtype not yet included in the WHO classification. Our findings suggest this rare tumor type may mainly affect adults in intra/juxta-ventricular locations, unlike their counterparts having the *BCOR* ITD alteration, thus constituting a novel diagnosis for neuropathologists to consider. As CNS tumors with *BCOR* ITD have been included in the chapter on embryonal tumors in the last WHO classification, further studies are needed to determine the cell of origin for their counterparts having *BCOR(L1)* fusions.

## Supplementary Information

**Additional file1: Figure S1**. Copy number variations plot from DNA-methylation analysis. A: Case #1. B: Case #2.
