# Supplementary figures and images for: CNS tumor with EP300::BCOR fusion: discussing its prevalence in adult population
Source: Acta Neuropathol Commun. 2023 Feb 13;11:26. doi: 10.1186/s40478-023-01523-y (PMC9926824; doi:10.1186/s40478-023-01523-y)

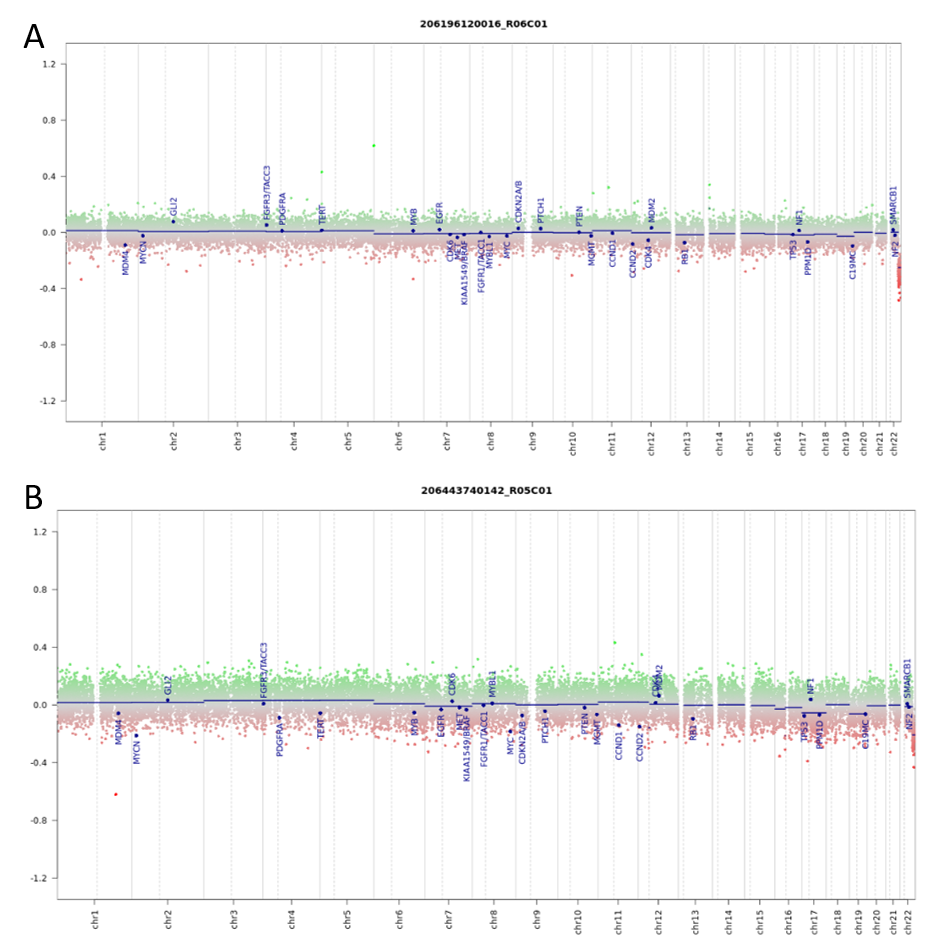

Supplement: Supplementary file 1 — Additional file1: Figure S1. Copy number variations plot from DNA-methylation analysis. A: Case #1. B: Case #2. [file 40478_2023_1523_MOESM1_ESM.tif]
